# Assessment of Practices in Chronic Hand Eczema Management From CHEMIN, a National Delphi Survey

**DOI:** 10.1111/cod.70001

**Published:** 2025-07-11

**Authors:** Claire Bernier, Marie‐Noëlle Crépy, Bruno Halioua, Camille Leleu, Marina Schverer, Solenn Le Clanche, Marie Tauber, Angèle Soria

**Affiliations:** ^1^ Service de Dermatologie et Plateforme Transversale D'allergologie Hôpital Hôtel Dieu, CHU Nantes Nantes France; ^2^ Service de Pathologie Professionnelle et Environnementale, Hôpital Hôtel Dieu AP‐HP; Service de Dermatologie Hôpital Cochin AP‐HP Paris France; ^3^ Centre Dermatologique Paris France; ^4^ Service de Dermatologie CHU Dijon‐Bourgogne Dijon France; ^5^ Public Health Expertise, Département Affaires Médicales Paris France; ^6^ Service D'allergologie et D'immunologie Clinique CIRI Inserm U1111, Hôpital Lyon Sud Lyon France; ^7^ Sorbonne Université, Service de Dermatologie et Allergologie Hôpital Tenon AP‐HP Paris France

**Keywords:** contact point, Delphi, eczema, questionnaires and surveys

Chronic Hand Eczema (CHE), affecting 4.9% of the French general population [1], is the leading occupational disease causing disability [2]. CHE management lacks standardised national guidelines, which may lead practitioners to rely on personal experience [3, 4]. We investigated, for the first time, French CHE diagnosis practices to assess ESCD guideline application.

## 1 Methods

A two‐round Delphi method survey, developed with six CHE experts, was distributed online from July 2023 to January 2024 through French medical societies. Targeted physicians were specialists in CHE, primarily dermatologists. Consensus was defined as agreement by > 66.6% of respondents. GDPR compliance and respondent confidentiality were ensured.

## 2 Results

**FIGURE 1:**
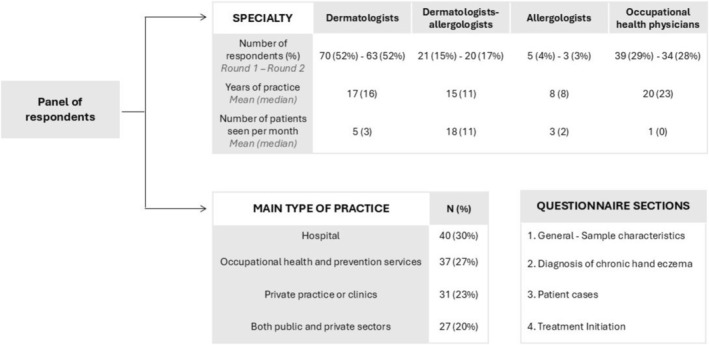
Characteristics of respondents and questionnaire sections. Respondents in round 2 were the same as in round 1.

A total of 135 respondents were analysed (Figure 1); 120 participated in round two. Most were dermatologists, with smaller proportions of dermatologists‐allergists, occupational health physicians, and allergists. Respondents saw ~6 CHE patients/month.

**FIGURE 2:**
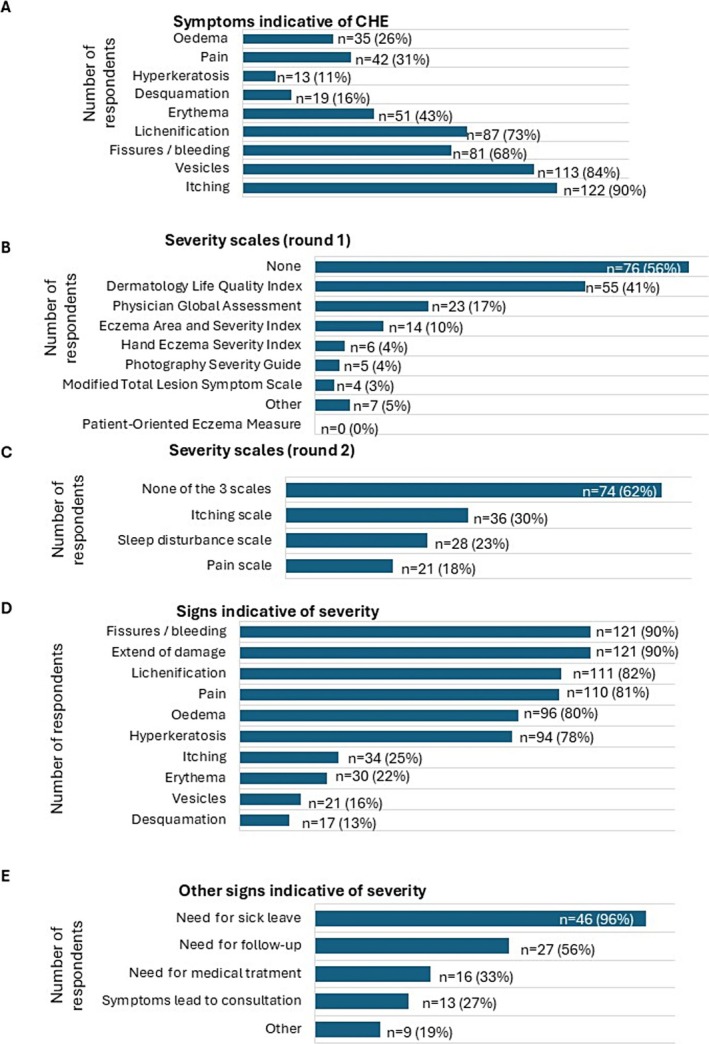
Assessment of clinical and functional symptoms indicative of CHE and CHE severity. (A) Clinical and functional symptoms indicative of CHE diagnosis. (B) Scales used by respondents to assess CHE severity (first round, Delphi method). (C) Scales used by respondents to assess CHE severity (second round, Delphi method). (D) Clinical and functional signs reported by respondent through the Delphi method. (E) Other signs considered as indicative of disease severity by 48 respondents in their clinical practice.

Consensus was reached on clinical signs of CHE: itch, vesicles, lichenification, and fissures/bleeding (Figure 2A). Hyperkeratosis, desquamation, oedema, and pain were not considered specific indicators.

No consensus emerged on standardised severity tools (Figure 2B,C). Among those using a severity scale, some used DLQI, whereas most relied on clinical signs (Figure 2B). Respondents identified clinical and functional severity signs (Figure 2D). Over one‐third included sick leave as additional criteria (Figure 2E).

Conditions in which allergology assessment is suggested (Table S1) and topics discussed with patients (Table S2) are not fully aligned. Notably, patients' quality of life (QoL) and food allergy were less frequently addressed.

## 3 Discussion

This Delphi, with a high response rate and strong representativeness [5], identified key areas for reflection to establish a clear alignment of essential steps to be implemented in France and to ensure better standardisation of patient care.

There was strong consensus on certain clinical signs as indicative of CHE, aligning with European guidelines [3]. Others were not but remained consistent with Yuksel et al. who found that combining ≥ 2 clinical signs with itching aids diagnosis [6].

The Delphi method had limitations, notably the limited input from allergists and, most importantly, the lack of consensus on severity scales—a critical finding of this study. Although some used DLQI, most based severity on specific clinical signs alone. Although European guidelines provide a comprehensive overview of these clinical and functional signs, our study presents new insights that could help improve severity diagnosis such as occupational incapacity. Besides, given that the severity reported by patients often exceeds the severity assessed by physicians, a standardised method could provide a more accurate assessment of CHE severity, potentially improving therapeutic decision‐making [6]. Standardising severity assessment, QoL evaluation, and key interview topics would improve practice, communication, and guideline alignment, highlighting the urgency for better CHE treatment.

## Author Contributions

**Claire Bernier:** conceptualization, investigation, writing – original draft, methodology, validation, writing – review and editing, formal analysis, resources. **Marie‐Noëlle Crépy:** conceptualization, investigation, writing – original draft, methodology, validation, writing – review and editing, formal analysis, resources. **Bruno Halioua:** conceptualization, investigation, writing – original draft, methodology, validation, writing – review and editing, formal analysis, supervision. **Camille Leleu:** conceptualization, investigation, writing – original draft, methodology, validation, writing – review and editing, formal analysis, supervision. **Marina Schverer:** writing – original draft, methodology, writing – review and editing, formal analysis, supervision, project administration. **Solenn Le Clanche:** writing – original draft, methodology, writing – review and editing, formal analysis, project administration, supervision. **Marie Tauber:** conceptualization, investigation, writing – original draft, methodology, validation, writing – review and editing, formal analysis, supervision. **Angèle Soria:** conceptualization, investigation, writing – original draft, validation, writing – review and editing, methodology, formal analysis, supervision.

## Ethics Statement

Confidentiality, GDPR compliance, and respondent anonymity were ensured by PHE, with no personal data shared with LEO Pharma French Affiliates or any third party. Regulatory statements were outlined on the first page of the questionnaire, and consent was obtained from respondents via a dedicated field before proceeding.

## Conflicts of Interest

C.B., M.‐N.C., B.H., C.L., M.T., and A.S. have disclosed conflicts of interest, including roles as consultants, speakers, advisory board members, or investigators for pharmaceutical companies such as Novartis, AbbVie, Lilly, LEO Pharma, Pfizer, and Sanofi. Additionally, M.T. reported consulting or speaking for Medac and receiving congress invitations from Sanofi, AbbVie, and Lilly, whereas A.S. disclosed similar invitations from Sanofi and Novartis. M.S. and S.L.C. authors declare no conflicts of interest.

## Supporting information


**Data S1.** cod70001‐sup‐0001‐Supinfo.
