# Supplementary material for: Assessment of Practices in Chronic Hand Eczema Management From CHEMIN, a National Delphi Survey
Source: Contact Dermatitis. 2025 Jul 11;93(4):336–8. doi: 10.1111/cod.70001 (PMC12415671; doi:10.1111/cod.70001)
Supplement: Supplementary file 1 — Data S1. cod70001‐sup‐0001‐Supinfo. [file COD-93-336-s001.docx]

**Online supplemental material**

**Table S1. Conditions in which allergology assessment is always, often, sometimes, rarely or never prescribed by respondents.** Percentage represents percentage of total respondents.

A consensus emerged on prescribing allergology assessments, primarily in cases where symptoms appear in the workplace and improve during vacations. However, fewer than 66% of respondents systematically prescribe it when patients are exposed to sensitizing, irritating, or allergenic substances, when a triggering factor is identified during questioning, or in cases of local treatment failure or resistance. Additionally, only 33% consider exacerbation of previously controlled lesions as an indication for allergology assessment. These findings underscore discrepancies in allergology referral practices and the need for a better communication on this matter to follow current guidelines.

|  | Always | Often | Sometimes | Rarely | Never |
| --- | --- | --- | --- | --- | --- |
| If symptoms appeared in the workplace and improved during vacation periods, % | 89 | 10 | 1 | 0 | 0 |
| If patient’s profession involve contact with sensitizing/irritating/allergenic substances, % | 65 | 31 | 3 | 1 | 0 |
| If the factor is identified during questioning, % | 64 | 26 | 6 | 2 | 2 |
| In case of failure or resistance to local treatment, % | 54 | 36 | 7 | 3 | 0 |
| In case of exacerbation of lesions previously controlled by local treatment, % | 33 | 44 | 19 | 3 | 1 |

**Table S2. Topics always, often, sometimes, rarely or never discussed during the initial or follow-up interviews with patients.** Percentage represents percentage of total respondents.

To evaluate how respondents diagnose CHE, they were asked about topics discussed during initial and follow-up consultations. Consensus was reached on assessing atopic dermatitis, asthma, rhino-conjunctivitis, atopic and psoriasis family history, leisure activities, soap use, exposure to irritants, glove use (type and material), and work-related QoL. However, no consensus emerged for food allergies, daily handwashing, or broader QoL assessments (personal, intimate, family, social, and sports-related). This highlights the limited consideration of personal QoL during CHE diagnosis and the need for greater alignment among specialists.

|  | Always | Often | Sometimes | Rarely | Never |
| --- | --- | --- | --- | --- | --- |
| Atopic dermatitis, % | 92 | 6 | 1 | 1 | 0 |
| Asthma, % | 87 | 10 | 1 | 1 | 1 |
| Rhino-conjunctivitis, % | 78 | 12 | 6 | 3 | 1 |
| Food allergy, % | 50 | 13 | 15 | 12 | 10 |
| Atopic family history, % | 74 | 16 | 4 | 2 | 4 |
| Psoriasis family history, % | 56 | 18 | 16 | 6 | 4 |
| Leisure activity, % | 82 | 14 | 4 | 0 | 0 |
| Daily handwashing, % | 58 | 24 | 10 | 3 | 5 |
| Soap used, % | 71 | 19 | 7 | 1 | 2 |
| Risk of contact with irritating substance, % | 86 | 11 | 3 | 0 | 0 |
| Gloves used when in contact with irritating substance, % | 91 | 7 | 2 | 0 | 0 |
| Types of gloves, % | 72 | 15 | 7 | 4 | 2 |
| Material of gloves, % | 71 | 18 | 5 | 2 | 4 |
| Quality of life – personal, intimate and family level, % | 42 | 37 | 15 | 3 | 3 |
| Quality of life – professional level, % | 73 | 23 | 3 | 1 | 0 |
| Quality of life – social level, % | 32 | 33 | 26 | 5 | 4 |
| Quality of life – leisure activity and sport, % | 29 | 34 | 19 | 5 | 13 |

**Supplementary References**

| **Section** | **Topic** | **Reference** |
| --- | --- | --- |
| **Introduction** | Absence of standardized national guidelines for CHE management | - Dubin C, Del Duca E, Guttman-Yassky E. Drugs for the Treatment of Chronic Hand Eczema: Successes and Key Challenges. Ther Clin Risk Manag. 2020;16:1319-1332. doi:10.2147/TCRM.S292504 - de León FJ, Berbegal L, Silvestre JF. Management of Chronic Hand Eczema. Actas Dermosifiliogr. 2015;106(7):533-544. doi:10.1016/j.ad.2015.04.005 - Lee GR, Maarouf M, Hendricks AK, Lee DE, Shi VY. Current and emerging therapies for hand eczema. Dermatol Ther. 2019;32(3):e12840. doi:10.1111/dth.12840 - Schuttelaar MLA. A new avenue for treatment of chronic hand eczema. Br J Dermatol. 2022;187(1):7-8. doi:10.1111/bjd.21604 - Hauber AB, Mohamed AF, Gonzalez JM, Otteson Fairchild A, Zelt SC, Graff O. Benefit-risk tradeoff preferences for chronic hand eczema treatments. J Dermatolog Treat. 2017;28(1):40-46. doi:10.1080/09546634.2016.1177161 |
| **Discussion** | Impact of a medical condition on quality of life and its role in guiding therapeutic decisions | - Skayem C, Salle R, Marquie A, et al. Perceived stress, disease burden and quality of life in patients with chronic hand eczema: A French national study. J Eur Acad Dermatol Venereol. 2024;38(6):e521-e524. doi:10.1111/jdv.19714 - Dalgard FJ, Gieler U, Tomas-Aragones L, et al. The psychological burden of skin diseases: a cross-sectional multicenter study among dermatological out-patients in 13 European countries. *J Invest Dermatol*. 2015;135(4):984-991. doi:10.1038/jid.2014.530 - Quaade AS, Alinaghi F, Dietz JBN, Erichsen CY, Johansen JD. Chronic hand eczema: A prevalent disease in the general population associated with reduced quality of life and poor overall health measures. *Contact Dermatitis*. 2023;89(6):453-463. doi:10.1111/cod.14407 |
